# Response to: the association between time definition of reintubation and patient outcomes in critically ill patients—several topics should be noticed

**DOI:** 10.1186/s13054-024-04817-2

**Published:** 2024-02-01

**Authors:** Aiko Tanaka, Tetsuhisa Kitamura, Akinori Uchiyama, Yusuke Enokidani, Yukiko Koyama, Takeshi Yoshida, Yuji Fujino

**Affiliations:** 1 Department of Anesthesiology and Intensive Care Medicine, Osaka University Graduate School of Medicine, 2-15 Yamadaoka, Suita, Osaka, 565-0871 Japan; 2 Department of Intensive Care, University of Fukui Hospital, Yoshida, Fukui, Japan; 3 Division of Environmental Medicine and Population Sciences, Department of Social and Environmental Medicine, Osaka University Graduate School of Medicine, Suita, Osaka, Japan

The authors appreciate Jiang and Zhang for their consideration of the topics regarding the association between reintubation and patient outcomes [1]. As discussed in our previous study [2] in *Critical Care*, reintubation involves multifactorial decisions. To evaluate the association between reintubation and patient outcomes, factors related to reintubation, in addition to mortality, should be determined as potential confounding factors. Therefore, we conducted the multivariable Cox proportional hazard analysis adjusted for age, sex, Acute Physiology and Chronic Health Evaluation III score (factors related to mortality), comorbidity of chronic heart failure and chronic respiratory failure, PaO_2_:FiO_2_, Glasgow Coma Scale score, and duration of first mechanical ventilation (factors related to reintubation). The multivariable analysis confirmed that reintubation was significantly associated with in-hospital and intensive care unit (ICU) mortality among the extubated patients and revealed novel findings that reintubation at 72–96 h after extubation was associated with the highest risk of mortality in critical care settings.

As noted by Jiang et al., patients who require prolonged mechanical ventilation or have difficulty weaning from mechanical ventilation undergo tracheotomy at the comprehensive decision of a clinician. Patients who underwent tracheostomy without an extubation attempt were not included in our analysis, and it is conceivable that the decision to perform tracheostomy may be affected by an inherent bias. A multicenter observational study showed significant differences in the timing of tracheostomy between ICUs [3]. Consequently, we performed a sensitivity analysis with additional adjustments for the participating sites in the multivariable models. Among all extubated patients, multivariable Cox proportional hazards analysis consistently described a significant association between reintubation and increased in-hospital and ICU mortality (adjusted hazard ratio [HR] 1.590, 95% confidence interval [CI], 1.418–1.783; *p* < 0.001 and adjusted HR 1.419, 95% CI, 1.139–1.770; *p* = 0.002, respectively). Regarding reintubated patients, multivariable analyses consistently demonstrated the highest in-hospital and ICU mortality rates in reintubated patients at 72–96 h after additional adjustment (see Table 1).

**Table 1:** Patient outcomes stratified by the timing of reintubation: Cox proportional hazards model with additional adjustment

	Reintubation ≤ 24 h	Reintubation 24–48 h	Reintubation 48–72 h	Reintubation 72–96 h	Reintubation 96–120 h
ICU mortality					
N (person-day)	74 (10,562)	38 (5540)	18 (2848)	15 (1692)	12 (1804)
Crude HR (95% CI)	1 (reference)	0.964 (0.651–1.428)	0.906 (0.540–1.519)	1.293 (0.741–2.256)	0.861 (0.462–1.604)
Adjusted HR (95% CI) *	1 (reference)	0.933 (0.603–1.443)	0.809 (0.461–1.419)	1.110 (0.593–2.077)	0.832 (0.427–1.619)
In-hospital mortality					
N (person-day)	183 (50,024)	96 (22,692)	53 (10,674)	35 (6467)	27 (5968)
Crude HR (95% CI)	1 (reference)	1.187 (0.9268–1.520)	1.385 (1.020–1.880)	1.524 (1.061–2.189)	1.303 (0.869–1.954)
Adjusted HR (95% CI) *	1 (reference)	1.193 (0.914–1.556)	1.218 (0.875–1.697)	1.499 (1.020–2.202)	1.279 (0.837–1.956)

*HR adjusted for age, sex, comorbidity of chronic heart failure, comorbidity of chronic respiratory failure, APACHE III score, PaO_2_:FiO_2_, Glasgow Coma Scale score, duration of first mechanical ventilation, use of noninvasive respiratory support, and site in mortality

*ICU*, Intensive care unit; *HR,* Hazard ratio; *CI,* Confidence interval; *APACHE,* Acute physiology and chronic health evaluation

The use of noninvasive respiratory support for post-extubation respiratory management has recently increased, and the decisions involved in mechanical ventilation, including the extubation process and criteria for reintubation and tracheostomy, have become multifaceted. Our findings, based on a large number of patients, were derived from a multicenter observational study. And the results were adjusted for numerous factors related to reintubation and mortality, with variations between sites, thus providing coherent validity and robustness. Consequently, the time definition of reintubation in terms of mortality identified in our study (72–96 h after extubation having the highest risk of death) has critical importance for the duration of observation of extubated patients in clinical practice as well as the uniformity of evidence in studies and guidelines. External validation of our findings, meta-analysis, and further investigations using randomized controlled trials are still required.

## Data Availability

The authors are contractually bound by the Japanese Intensive Care PAtient Database (JIPAD) project to avoid publishing or sharing the data used in this manuscript.

## Abbreviations

CI: Confidence interval
HR: Hazard ratio
ICU: Intensive care unit
